# Involvement of the Dectin-1 Receptor upon the Effector Mechanisms of Human Phagocytic Cells against *Paracoccidioides brasiliensis*

**DOI:** 10.1155/2019/1529189

**Published:** 2019-02-06

**Authors:** Juliana Carvalho de Quaglia e Silva, Amanda Manoel Della Coletta, Taiane Priscila Gardizani, Graziela Gorete Romagnoli, Ramon Kaneno, Luciane Alarcão Dias-Melicio

**Affiliations:** ^1^São Paulo State University (UNESP), Medical School of Botucatu, Laboratory of Immunopathology and Infectious Agents - LIAI, UNIPEX - Experimental Research Unity, Sector 5, Botucatu SP, Brazil; ^2^São Paulo State University (UNESP), Institute of Biosciences, Department of Microbiology and Immunology, Botucatu SP, Brazil; ^3^São Paulo State University (UNESP), Medical School of Botucatu, Department of Pathology, Botucatu SP, Brazil

## Abstract

Paracoccidioidomycosis (PCM), a systemic mycosis endemic in Latin America, occurs after inhalation of mycelial components of *Paracoccidioides* spp. When the fungus reaches the lungs and interacts with the alveolar macrophages and other cells, phagocytic cells such as neutrophils and monocytes are immediately recruited to the injured site. The interaction between surface molecules of pathogens and homologous receptors, present on the surface membrane of phagocytes, modulates the innate immune cell activation. Studies have shown the importance of fungal recognition by the Dectin-1 receptor, which can induce a series of cellular protective responses against fungi. The objective of the present study was to evaluate Dectin-1 receptor expression and the effector mechanisms of human monocytes and neutrophils activated or not with different cytokines, such as IFN-*γ*, TNF-*α*, and GM-CSF, followed by the challenge with *Paracoccidioides brasiliensis* (*P*. *brasiliensis* or Pb265). Therefore, analysis of Dectin-1 receptor expression was done by flow cytometry whereas the effector mechanisms were evaluated by fungal recovery by colony-forming unit (CFU) counting and hydrogen peroxide (H_2_O_2_) production. Our results showed that, after treatment with IFN-*γ*, TNF-*α*, and GM-CSF and challenge with Pb265, cells, especially monocytes, demonstrated an increase in Dectin-1 expression. Both types of cells treated with the cytokines exhibited a decreased fungal recovery and, conversely, an increased production of H_2_O_2_. However, when cultures were treated with an anti-Dectin-1 monoclonal antibody, to block the *P*. *brasiliensis* binding, a decrease in H_2_O_2_ production and an increase in fungal recovery were detected. This effect was observed in all cultures treated with the specific monoclonal antibody. These results show the involvement of the Dectin-1 receptor in fungal recognition and its consequent participation in the induction of the killing mechanisms against *P*. *brasiliensis*.

## 1. Introduction

Paracoccidioidomycosis (PCM) is a systemic mycosis endemic in Latin America, especially in some regions of Brazil [1]. The etiologic agents of PCM are the fungi encompassed in the genus *Paracoccidioides* [2], which are thermodimorphic fungi that are presented as mycelium at room temperature ranging from 4 to 28°C and also grow as yeast *in vivo*, in host tissues or *in vitro* cultures at 37°C in enriched culture media. The infection occurs by the inhalation of conidia or mycelial components which reach the lungs causing local lesions or can disseminate to other organs by lymphatic or blood vessels [3].

At the beginning of the infection, neutrophils and monocytes are recruited to the injured site. The fungus is initially recognized by Pattern Recognition Receptors (PRRs), and among them, the Dectin-1 receptor, a member of the C-type lectin-like receptors (CLR) can bind specifically to *β*-1,3 glucans in the fungal cell wall [4–9]. The latest studies have characterized the different activation pathways of the Dectin-1 receptor in response to *P*. *brasiliensis* that triggers cellular activation leading to a modulatory function through the production of chemokines and cytokines such as TNF-*α*, IL-1*β*, IL-18, IL-12, IL-8, IL-17, and IL-10 [9–11].

The treatment of human neutrophils and monocytes or murine macrophages with proinflammatory cytokines like IFN-*γ*, TNF-*α*, and GM-CSF has been widely studied in response to many fungal species, including *P*. *brasiliensis*. The priming of cells with these cytokines is responsible for enhancement of phagocytosis, antifungal activity, and oxidative burst [12–18]. However, there are no studies evaluating the expression of the Dectin-1 receptor by cells after activation with IFN-*γ*, TNF-*α*, and GM-CSF, the possible relation with the production of hydrogen peroxide (H_2_O_2_), and the fungicidal activity of phagocytic cells against *P*. *brasiliensis*.

In this study, we demonstrated the involvement of the Dectin-1 receptor on *P*. *brasiliensis* (Pb265) killing and H_2_O_2_ production by human neutrophils and monocytes preactivated with human recombinant cytokines, IFN-*γ*, TNF-*α*, and GM-CSF.

## 2. Materials and Methods

### 2.1. Study Group

Neutrophils and monocytes were obtained from 40 mL of peripheral blood of eight healthy volunteer donors (both gender) by venipuncture. Each subject signed the consent form after explanation of the research objectives. This study was conducted according to the principles expressed in the Declaration of Helsinki and was approved by the Research Ethics Committee of Botucatu Medical School, UNESP (São Paulo State University) (Of. 526/2012).

### 2.2. *P*. *brasiliensis* Preparation

This study used the strain 265 of *P*. *brasiliensis* (Pb265), which has higher amounts of *β*-glucan on its walls [19, 20], kept in our laboratory by culture in a GPY medium (1.5% glucose, 1% peptone, and 0.5% yeast extract) at 37°C for 6 days of cultivation. After growth, yeast cells were transferred to sterile tubes containing glass beads and approximately 10 mL of a RPMI-1640 tissue culture medium (Sigma-Aldrich, St. Louis, USA) and were mixed in a vortex homogenizer for 30 seconds. To determine the yeast viability, phase-contrast microscopy was used, since viable cells presented a bright appearance (refractive) and dead cells showed a dark wall [21]. Fungal suspensions with at least 95% of viability were used in this study. For flow cytometry assay, fungicidal activity, and H_2_O_2_ measurement, the Pb265 concentration was adjusted establishing a challenge rate of fungus/monocytes or neutrophils of 1 : 50 ratio.

### 2.3. Isolation and Culture of Neutrophils and Monocytes from Peripheral Blood

Blood sample was collected by a BD Vacutainer system and vacuum-heparinized tubes (BD Biosciences, San Diego, CA, EUA). Neutrophils and monocytes were separated by a density gradient centrifugation: Histopaque® 1.119 g/mL and above Histopaque® 1.083 g/mL (both from Sigma-Aldrich) at 1500 rpm for 30 minutes, at room temperature (25°C). Only neutrophils underwent an erythrocyte lysis with a hypotonic solution (NaCl 0.2%). Cell viability and neutrophil counting were evaluated by trypan blue staining, considering viability greater than or equal to 95%. Neutral red staining (0.02%) was also used for monocyte counting.

For flow cytometry analysis, cell concentrations were adjusted to 1 × 10^6^ cells/mL. To evaluate the fungal recovery and production of hydrogen peroxide, both cell types were adjusted for 2 × 10^6^ cells/mL. Monocyte cultures were incubated for 2 hours prior to the treatment, at 37°C, in a 5% CO_2_ atmosphere, for the adhesion of these cells on the bottom of the wells. After, the wells were washed twice with the culture medium in order to remove nonadherent cells.

### 2.4. Cell Treatment with Cytokines, Blockage of the Dectin-1 Receptor, and Challenge with *P*. *brasiliensis*

Neutrophil and monocyte cultures were activated or not with the following cytokines: IFN-*γ* (250 U/mL), TNF-*α* (500 U/mL), and GM-CSF (250 U/mL) for 18 h before the challenge with the fungus. All recombinant cytokines were purchased from the R&D Systems, Inc. (Minneapolis, MN).

In some cultures, two hours before the challenge with the fungus, cells were incubated or not with 10 *μ*L (3 *μ*g/mL) of anti-Dectin-1 monoclonal antibody (monoclonal anti-human Dectin-1/CLEC7A antibody, R&D Systems), to block the receptor and consequently evaluate the Dectin-1 role in *P*. *brasiliensis* recognition, fungicidal activity, and H_2_O_2_ production by both types of cells. The analysis of the receptor blockage was done by flow cytometry. Control groups with only the monoclonal antibody were previously tested, showing no direct effect on the evaluated mechanisms.

After the treatments and receptor blocking protocol, monocytes and neutrophils were challenged with *P*. *brasiliensis* (Pb265) for 4 hours. All procedures were carried out at 37°C, in a 5% CO_2_ atmosphere.

### 2.5. Dectin-1 Expression on the Surface of Neutrophils and Monocytes by Flow Cytometry

After the neutrophil and monocyte treatment or not with cytokines, blockage of receptor, and challenge with *P. brasiliensis*, cells were recovered by addition of 1 mL of Isoton (BD Biosciences) and transferred to flow cytometric tubes. The tubes were centrifuged, the supernatant was discharged, and the monocytes were incubated with 10 *μ*L of anti-CD_14_^+^ antibody conjugated with FITC fluorochrome (BD Biosciences), while the neutrophils were incubated with 3 *μ*L of anti-CD_16_^+^ antibody conjugated with PerCP (BioLegend CNS, Inc. San Diego, CA), as markers of cell populations, according to the manufacturers' instructions. Both types of cells were also incubated with 3 *μ*g/mL of anti-Dectin-1 antibody conjugated with PE fluorochrome (AbD Serotec, Bio-Rad, Kidlington, UK). Data were acquired using the flow cytometry FACSCanto II model with the FACSDiva software, from the Microbiology and Immunology Department of the Botucatu Biosciences Institute, UNESP. Cellular multiple parameters were analyzed by the FlowJo software. The standard acquisition was set to 10,000 events and it optimized the population of interest by establishing a gate based in size (Forward-Scattered, FSC), granularity (Side-Scattered, SSC), and fluorescence parameters.

### 2.6. Fungal Recovery

After cytokine treatment and Dectin-1 receptor blockage, neutrophils and monocytes were challenged with Pb265 for 4 h. The coculture supernatants were collected and the wells were washed many times with sterile distilled cold water to remove and lyse the cells and consequently release the phagocyted fungi. The solution obtained was considered to be experimental cultures. Cultures containing only the fungus were used as a control. Each experimental and control culture well washing resulted in a final volume of 2 mL, and 100 *μ*L was added (in duplicate) on petri dishes containing the brain–heart infusion (BHI) agar medium (Oxoid Ltd. England) supplemented with 4% horse serum, 50 *μ*g/mL of gentamicin, and 5% *P*. *brasiliensis* strain 192 culture filtrate (*v*/*v*), which constituted the source of the growth-promoting factor [22]. Experimental and control plates were incubated at 37°C, and after 10 days, the number of colony-forming units (CFU) per plate was counted. The fungal recovery percentage was determined by the following formula:

(1) $$\begin{array}{c} \%\text{ fungal recovery}=\left[\frac{\text{experimental culture CFU mean}}{\text{control culture CFU mean}}\right]\times 100 \end{array}$$

### 2.7. H_2_O_2_ Production

H_2_O_2_ production was determined by the horseradish peroxidase-phenol red oxidation method used by Carmo et al. [15]. For this, after the challenge, culture supernatants were removed and 100 *μ*L of phenol red solution (140 mM of NaCl; 10 mM phosphate buffer pH 7; 5.5 mM dextrose; 0.56 mM phenol red; 0.01 mg/mL horseradish peroxidase type II) (Sigma Chemical Co. St. Louis, MO, USA) was added in each well. After 1 hour, the reaction was interrupted by adding 10 *μ*L of NaOH 1N. Absorbance was measured in an automatic ELISA reader with a 620 nm filter. Results were expressed in nanomoles and compared to an established standard curve for each assay in accordance with the following concentrations of 0.5, 1.0, 2.0, 4.0, and 8.0 nM H_2_O_2_.

### 2.8. Statistical Analysis

Dectin-1 receptor expression and fungal recovery data were analyzed by the ANOVA test, followed by the Tukey-Kramer test, with a significance level of *p* < 0.05. H_2_O_2_ production data were analyzed by the ANOVA test, followed by Dunn's multiple comparison test, with a significance level of *p* < 0.05. All tests were performed using the software SigmaPlot version 12.0 (Softonic).

## 3. Results

### 3.1. IFN-*γ*, TNF-*α*, and GM-CSF Action and *P*. *brasiliensis* Challenge on Dectin-1 Receptor Expression in Monocytes and Neutrophils

**Figure 1:**
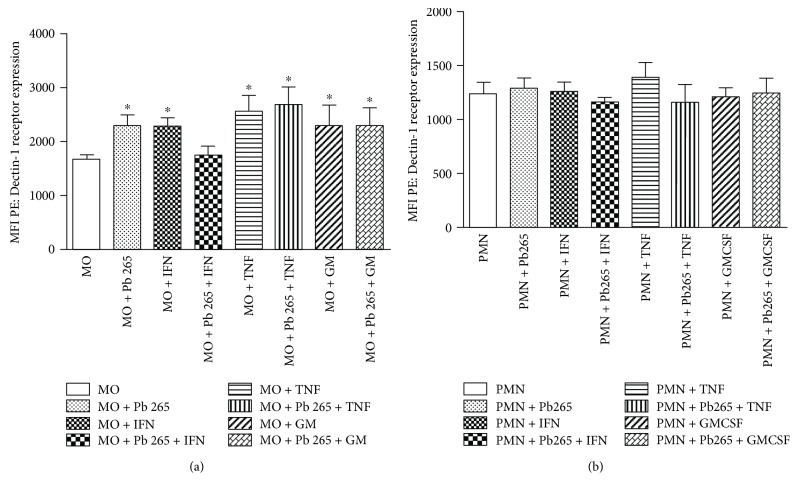
Dectin-1 receptor expression (MFI) of monocytes (MO) (a) and neutrophils (PMN) (b) treated or not with IFN-*γ*, TNF-*α*, and GM-CSF for 18 hours followed by the Pb265 challenge or not for 4 hours. Data are expressed as the mean of 8 healthy volunteer donors tested. ^∗^Statistical significance between groups is indicated (^∗^*p* < 0.05 × MO control).

Our data demonstrated that CD_14_^+^ monocytes showed increased Dectin-1 receptor expression when cells were treated with IFN-*γ*, TNF-*α*, and GM-CSF. The challenge with Pb265 for 4 hours, with or without preactivation of these cells with TNF-*α* and GM-CSF, also caused an increase in receptor expression (Figure 1(a)).

When evaluating the CD_16_^+^ neutrophil cultures treated with IFN-*γ*, TNF-*α*, and GM-CSF, followed by the challenge with Pb265, we did not identify significant differences between groups (Figure 1(b)).

We also observed that the mean fluorescence intensity (MFI) detected in the assays was higher in the CD_14_^+^ monocyte cultures (Figure 1(a)) than in the CD_16_^+^ neutrophil cultures (Figure 1(b)), demonstrating a greater expression of this receptor by monocytes, although the percentage of positive cells for the Dectin-1 receptor was similar in both cell types (data not shown).

### 3.2. Dectin-1 Receptor Role in the Fungal Recovery from Monocytes and Neutrophils

**Figure 2:**
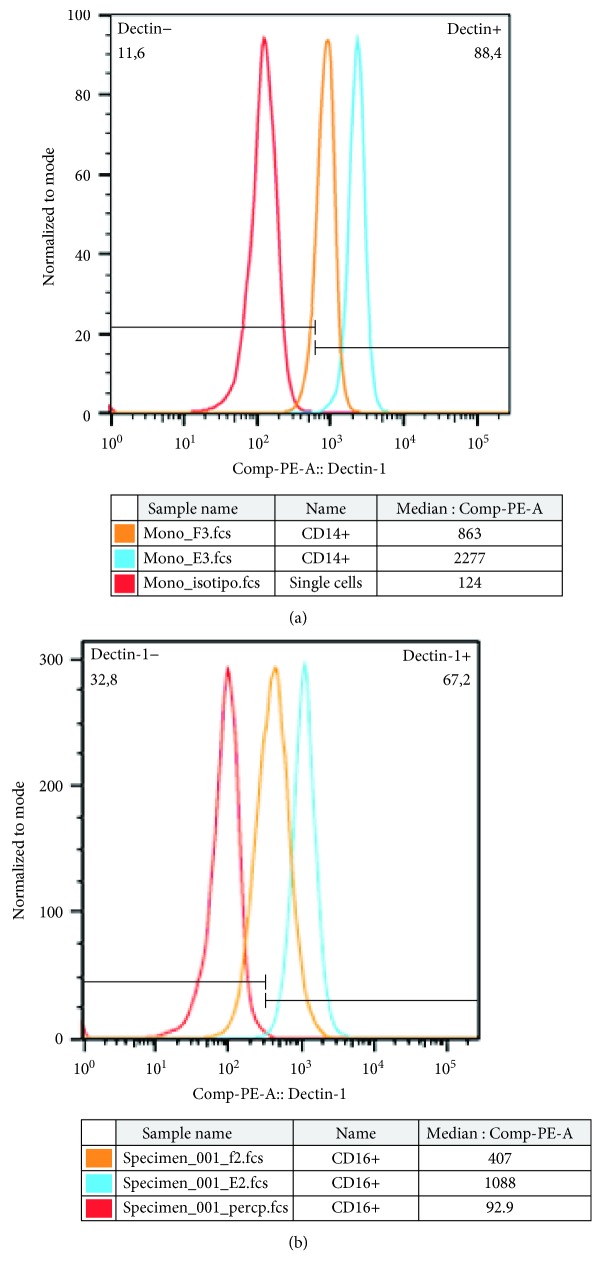
Histogram of a representative experiment for Dectin-1 receptor blockage for monocytes (a) and neutrophils (b), showing MFI of monocytes or neutrophil cultures (blue line) or monocytes or neutrophils treated with 3.0 *μ*g/mL (orange line) of anti-Dectin-1 monoclonal antibody. The red line represents MFI of the isotypic control.

**Figure 3:**
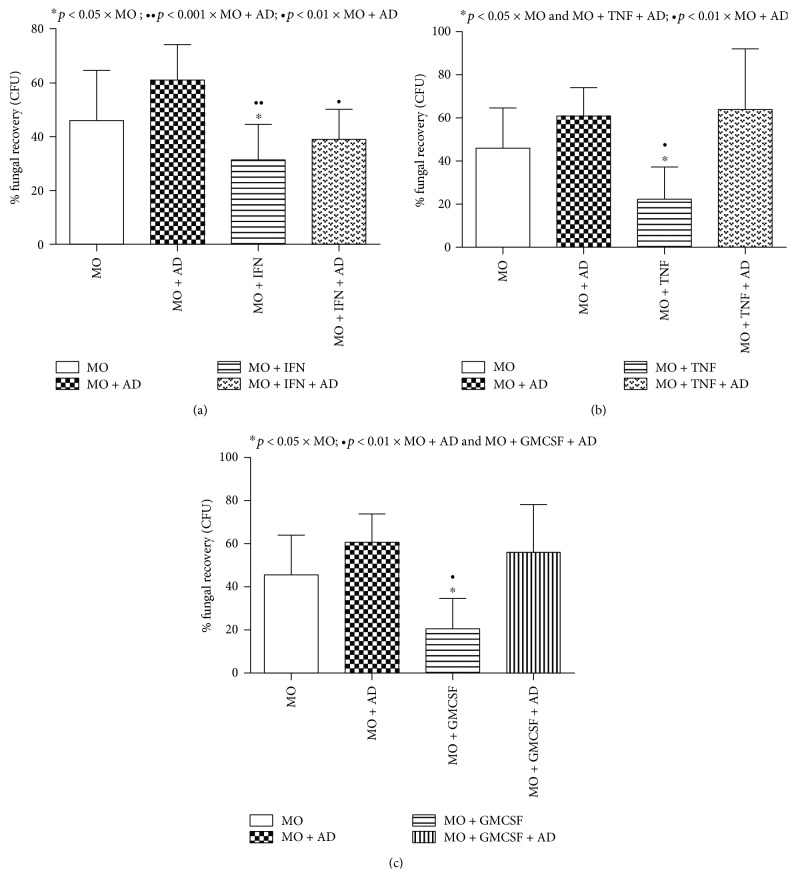
Percentage of fungal recovery by colony-forming unit (CFU) analysis of monocyte (MO) cultures treated or not with IFN-*γ* (a), TNF-*α* (b), and GM-CSF (c) for 18 hours in the presence or absence of the anti-Dectin-1 monoclonal antibody (AD) and challenged with Pb265 for 4 hours. Data are expressed as the mean of 8 healthy volunteer donors tested. Statistical significance between groups is indicated.

To assess the participation of the Dectin-1 receptor on fungal recovery from phagocytic cells, we used blockage protocol with the anti-Dectin-1 monoclonal antibody, achieving a rate of 53% and 46% blockage of monocyte and neutrophil receptors, respectively (Figures 2(a) and 2(b)). As observed in Figures 3(a)–3(c), our results demonstrated that all cytokines (IFN-*γ*, TNF-*α*, and GM-CSF) were capable of reducing fungal recovery in monocyte cultures. The treatment with the anti-Dectin-1 monoclonal antibody induced a fungal recovery increase in the treated groups, indicating the receptor's participation in the monocyte killing mechanism against *P. brasiliensis*.

**Figure 4:**
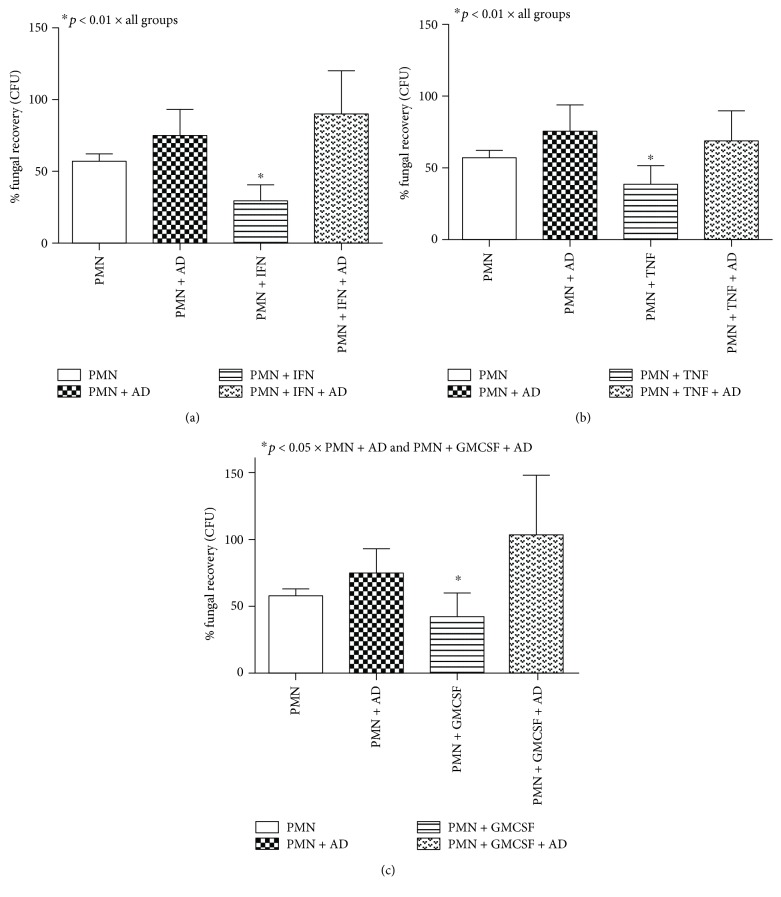
Percentage of fungal recovery by colony-forming unit (CFU) analysis of neutrophil (PMN) cultures treated or not with IFN-*γ* (a), TNF-*α* (b), and GM-CSF (c) for 18 hours in the presence or absence of the anti-Dectin-1 monoclonal antibody (AD) and challenged with Pb265 for 4 hours. Data are expressed as the mean of 8 healthy volunteer donors tested. Statistical significance between groups is indicated.

When analyzing neutrophil data in Figure 4, we identified similar results to monocytes. The treatment with IFN-*γ*, TNF-*α*, and GM-CSF cytokines was capable of reducing fungal recovery from the cocultures (Figures 4(a)–4(c), respectively). Besides, the Dectin-1 receptor blockage also induced an increase in the fungal recovery from these cultures (Figure 4), showing the same effect observed in the monocyte cultures.

### 3.3. Dectin-1 Receptor Involvement on the H_2_O_2_ Production by Monocytes and Neutrophils

**Figure 5:**
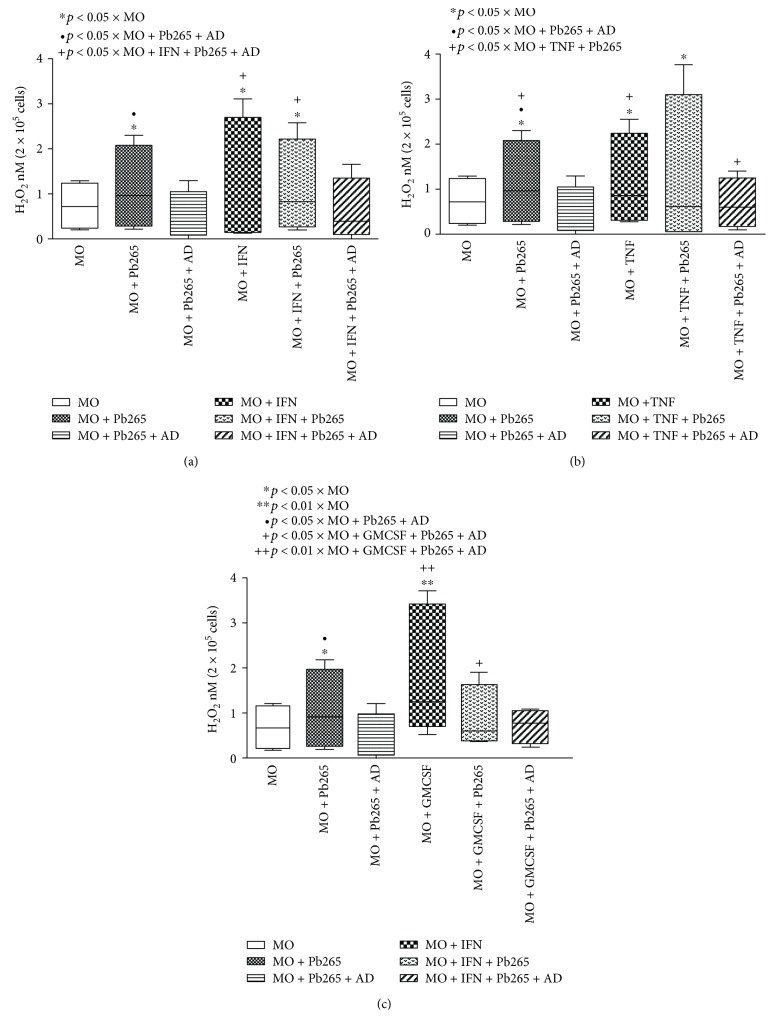
Hydrogen peroxide (H_2_O_2_) production by monocytes (MO) treated or not with IFN-*γ* (a), TNF-*α* (b), and GM-CSF (c) for 18 hours in the presence or absence of the anti-Dectin-1 monoclonal antibody (AD) and challenged or not with Pb265 for 4 hours. Box-and-whisker plot showing data distribution of 8 healthy volunteer donors. Statistical significance between groups is indicated.

Our results demonstrated that monocytes challenged with Pb265 showed an increase in the H_2_O_2_ production (Figure 5). In monocyte cultures treated with IFN-*γ*, TNF-*α*, and GM-CSF and challenged or not with Pb265, we also identified an increase of a metabolic product when compared with the control cultures (Figures 5(a)–5(c), respectively). However, the production of this reactive oxygen product decreased when the challenged cultures were treated with the specific anti-Dectin-1 monoclonal blocking antibody.

**Figure 6:**
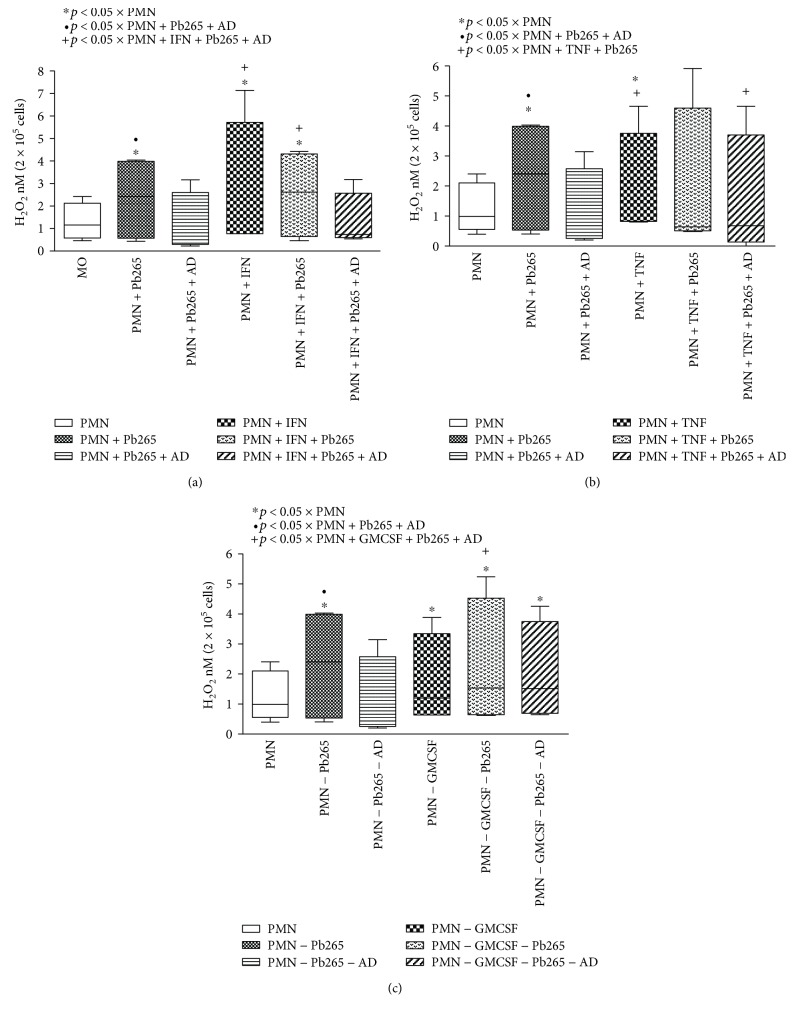
Hydrogen peroxide (H_2_O_2_) production by neutrophils (PMN) treated or not with IFN-*γ* (a), TNF-*α* (b), and GM-CSF (c) for 18 hours in the presence or absence of the anti-Dectin-1 monoclonal antibody (AD) and challenged or not with Pb265 for 4 hours. Box-and-whisker plot showing data distribution of 8 healthy volunteer donors. Statistical significance between groups is indicated.

Neutrophil cultures showed similar results to monocytes. When treated with IFN-*γ*, TNF-*α*, and GM-CSF and challenged or not with Pb265, neutrophils also demonstrated an increase in H_2_O_2_ levels in relation to the control cultures (Figures 6(a)–6(c), respectively). The receptor blockage also resulted in decreased levels of H_2_O_2_ in cultures treated with the blocking antibody.

## 4. Discussion

An effective antifungal immunity involves different PRRs which can recognize many components of the fungal cell wall, giving a synergistic effect on cell activation [23]. Several studies have shown the role of the Dectin-1 receptor as a prominent activator of phagocytosis and respiratory burst (reactive oxygen species production) in phagocytes and in the production of inflammatory mediators in fungal infections [24–31]. However, the role of this receptor in human phagocytes is still uncertain, since the studies present conflicting data [32–34].

In PCM, many studies have contributed to the understanding of Dectin-1 receptor functions in human phagocytes. Studies that evaluated the participation of the receptor used different protocols, demonstrating results such as receptor participation in fungal recognition, induction of effector mechanisms, and production of cytokines such as TNF-*α* [4, 6, 7, 9–11, 35–37]. In experimental models of the disease, there are conflicting data showing the participation of the Dectin-1 receptor in susceptibility and in PCM resistance [5, 8, 11, 38–40].

Our results showed that the challenge with Pb265 and the treatments with IFN-*γ*, TNF-*α*, and GM-CSF induced an increase in Dectin-1 receptor expression in monocytes similarly. These treatments also led to a decrease in the viable fungal recovery in these cultures and an increased production of H_2_O_2_. However, this effect was reversed when the binding of *P*. *brasiliensis* to the Dectin-1 receptor was blocked with the anti-Dectin-1 monoclonal blocking antibody, identifying a decrease in H_2_O_2_ production and an increase in fungal recovery.

Previous studies from our group showed that a preincubation of monocytes with IFN-*γ* did not induce a more efficient fungicidal activity against a virulent strain of *P*. *brasiliensis* (Pb18). This mechanism was only improved when a preincubation with TNF-*α*, TNF-*α* plus IFN-*γ*, or GM-CSF was made [12, 15, 41]. When cells were challenged with Pb265 (considered a less virulent strain), the preactivation with IFN-*γ* alone was enough to acquire an efficient fungicidal activity [12].

In relation to neutrophils, we obtained similar results to those observed for monocytes. Treatment with IFN-*γ*, TNF-*α*, and GM-CSF also led to a decreased recovery of viable fungi from cell cultures and an increased production of H_2_O_2_ levels, higher than those produced by monocytes. This effect was also reversed with the blockage of the *P*. *brasiliensis* binding to the receptor by treating the cultures with the anti-Dectin-1 monoclonal antibody.

The requirement of neutrophil activation for the *P*. *brasiliensis* killing has also been observed in some studies. Studies have shown that activated human neutrophils did not have neither fungistatic nor fungicidal activity against *P*. *brasiliensis* [42]. These antifungal effects were significantly increased upon activation with IFN-*γ*, IL-1, and GM-CSF, but TNF-*α* and IL-8 cytokines had no effect on these activities [42–44]. Our group, evaluating the effect of cytokines such as IFN-*γ*, TNF-*α*, GM-CSF [16], and IL-15 [45] on human neutrophil fungicidal activity against *P*. *brasiliensis,* showed that nonactivated neutrophils did not develop antifungal activity. However, a significant fungicidal activity was obtained after incubation with IFN-*γ*, TNF-*α*, GM-CSF, and IL-15. Additionally, the results demonstrated the involvement of superoxide anion and H_2_O_2_ as effector molecules of activated neutrophils against the fungus.

However, although Dectin-1 receptor blockage with the specific monoclonal antibody resulted in a decrease of H_2_O_2_ production and increased fungal recovery, we observed no difference in Dectin-1 receptor expression in neutrophils, with a smaller mean fluorescence intensity (MFI) than those presented by the monocyte cultures.

We could have expected that activated and challenged cells would exhibit enhanced receptor expression, demonstrating a synergistic effect between the cytokines and the fungus. However, we believe that, in our conditions, the maximum expression of this receptor by the cells could be reached; once in previous study [9], we showed that, in the same conditions, IFN-*γ* treatment induced monocytes to produce TNF-*α*, and this production was higher when cells were activated with IFN and challenged with Pb265, being TNF secretion by monocytes was induced via the Dectin-1 receptor. We also showed that GM-CSF treatment induced TNF-*α* production by monocytes, and this production was also higher when cells were activated with GM-CSF and challenged with Pb265, and all these could account to the maximum receptor expression. Regarding neutrophils, it seems that they already express the maximum amount of receptors and that they are not modulated by these cytokines or by the challenge with the fungus. This could be an important mechanism of these cells, which guarantees the recognition of the fungus independent of the cytokine environment present. Besides, in other study [36], we showed another important extracellular killing mechanism demonstrating the participation of the Dectin-1 receptor on NET (Neutrophil Extracellular Trap) release against *Paracoccidioides brasiliensis,* which would be acting jointly with the H_2_O_2_, the metabolite involved in the destruction of the fungus.

Thus, our study demonstrated that the induction of an intense inflammatory response observed in previous studies [12, 15, 16, 41] could be mediated through a preferential binding of the fungus to the Dectin-1 receptor, which is expressed on the phagocyte membrane. This interaction triggers the effector mechanisms against the fungus, such as H_2_O_2_ production, resulting in lower fungal recovery. These results also provided new knowledge, demonstrating differences between the both types of cells and also indicating that Dectin-1 receptor expression in neutrophils does not undergo modulation by these cytokines nor after the *P. brasiliensis* challenge.

## Data Availability

The data used to support the findings of this study are included within the article.
